# Development of a Novel Simulation Reactor for Chronic Exposure to Atmospheric Particulate Matter

**DOI:** 10.1038/srep42317

**Published:** 2017-02-07

**Authors:** Jianhuai Ye, Sepehr Salehi, Michelle L. North, Anjelica M. Portelli, Chung-Wai Chow, Arthur W. H. Chan

**Affiliations:** 1 Department of Chemical Engineering & Applied Chemistry, University of Toronto, Canada; 2 Division of Respirology and Multi-Organ Transplantation Programme, University Health Network, University of Toronto, Canada; 3 Dalla Lana School of Public Health, University of Toronto, Canada.

## Abstract

Epidemiological studies have shown that air pollution is associated with the morbidity and mortality from cardiopulmonary diseases. Currently, limited experimental models are available to evaluate the physiological and cellular pathways activated by chronic multi-pollutant exposures. This manuscript describes an atmospheric simulation reactor (ASR) that was developed to investigate the health effects of air pollutants by permitting controlled chronic *in vivo* exposure of mice to combined particulate and gaseous pollutants. BALB/c mice were exposed for 1 hr/day for 3 consecutive days to secondary organic aerosol (SOA, a common particulate air pollutant) at 10–150 μg/m^3^, SOA (30 μg/m^3^) + ozone (65 ppb) or SOA + ozone (65 ppb) + nitrogen dioxide (NO_2_; 100 ppb). Daily exposure to SOA alone led to increased airway hyperresponsiveness (AHR) to methacholine with increasing SOA concentrations. Multi-pollutant exposure with ozone and/or NO_2_ in conjunction with a sub-toxic concentration of SOA resulted in additive effects on AHR to methacholine. Inflammatory cell recruitment to the airways was not observed in any of the exposure conditions. The ASR developed in this study allows us to evaluate the chronic health effects of relevant multi-pollutant exposures at ‘real-life’ levels under controlled conditions and permits repeated-exposure studies.

Exposure to air pollution, including airborne particulate matter (PM) and gaseous pollutants, such as ozone (O_3_) and nitrogen dioxide (NO_2_), increases the incidence, morbidity and mortality from multiple diseases^1^,^2^,^3^. Extensive epidemiological evidence has shown the strongest link between exposure to air pollutants and increased burden of cardiopulmonary diseases, such as myocardial infarction^4^, asthma^5^ and chronic obstructive pulmonary disease^6^.

Air pollution is a complex mixture of PM and gases, whose chemical composition depends on the source emissions, and also changes over time as a result of atmospheric processing. PM has received increasing attention over the past decades as multiple studies have observed strong associations between PM exposures and increased hospital admissions for and death from cardiorespiratory illnesses^7^,^8^, while decreases in ambient PM levels have been associated with increased longevity^9^,^10^.

Epidemiological studies are useful for assessing the effects of real-life exposure, but they need to be complemented by toxicological studies to establish direct causal relationships and to identify physiological pathways and cellular mechanisms. Currently, these studies have primarily been confined to *in vitro* exposures using cell lines or short-term acute *in vivo* exposure studies with animal models. PM-induced oxidative stress and the ensuing inflammation have emerged from *in vitro* studies as an important paradigm in the pathogenesis of PM-induced toxicity^5^. However, there is emerging evidence that other pathways activated by pollutants, such as DNA modification^11^ and altered immune responses^12^ are also important. Moreover, studies have clearly shown that pollutant-mediated cellular pathways occur in the absence of oxidative stress^13^,^14^,^15^, suggesting that non-inflammatory pathways leading to negative health outcomes should also be considered.

The use of mouse and rat models for *in vivo* exposure studies have greatly expanded our understanding of the pathways involved in PM-mediated responses. To date, exposure of small animals to diesel/gasoline exhaust, concentrated ambient PM and other pollutants have been carried out using two main techniques: (1) intranasal instillation of PM into animal lungs^16^, and (2) exposure chambers with PM from on-line generation or concentrated ambient air with particulates alone or in combination with gases^17^,^18^,^19^. However, both methods have shortcomings. The distribution of PM deposition following intranasal instillation in the lung may be different from the physiological inhalation route^16^,^19^. On-line PM generation in previous exposure chamber studies usually generates an atmosphere of “raw exhaust” that is not normally encountered by the general population, except in some occupational scenarios. Of the available systems, concentrated ambient PM may represent the most relevant mixture of particulates to model street-level exposures in humans. However, infrastructure requirements include specialized equipment for concentration of ambient PM and the attendant human resources and expertise to operate the equipment, and thus have limited the availability of such research facilities to a few laboratories worldwide.

Reproducing ambient PM in controlled and consistent conditions for experimentation is also challenging. The composition is highly dependent on local source emissions and is subject to spatial and temporal variations. The composition of the organic fraction, which constitutes 20–90% of submicron particle mass, can also change dynamically as a result of atmospheric processing^20^. For example, a major constituent of ambient PM is formed from oxidation of organic gases, leading to condensation of low-volatility products, known as secondary organic aerosol (SOA)^22^. It is postulated that SOA may be more toxic as it has higher oxidative potentials *in vitro*^23^ and generates greater oxidative stress *in vivo*, but a direct causal relationship has not yet been identified.

In this study, we developed an atmospheric simulation reactor (ASR) that permits controlled exposures of mice to different combinations and concentrations of PM (e.g. SOA) and gaseous pollutants. The ASR is small and portable. Thus, the unit can be installed easily within most research animal facilities for studies of repeated exposures over days to weeks. Together, these features allow us to employ the ASR for chronic *in vivo* exposures to relevant pollutants at “real-life” level to interrogate the mechanisms by which pollution causes disease. To verify the feasibility of our ASR, the pulmonary response in healthy BALB/c mice exposed to SOA from naphthalene oxidation (N-SOA) was examined. Naphthalene was chosen as the SOA precursor because it is the most abundant polycyclic aromatic hydrocarbon (PAH) emitted from anthropogenic activities^24^. Thus, it is highly relevant for studying the toxicity of PM from urban emissions. The composition of N-SOA is also well constrained, allowing us to explore the associations between specific SOA species and toxicities. We demonstrated efficient delivery to the lungs of well-characterized N-SOA, and increased airway hyperresponsiveness following exposure to SOA in a dose-dependent manner, as well as an additive effect of exposure to multi-pollutant atmospheres consisting of mixtures of both N-SOA and gases.

## Results

### Efficient delivery of SOA to mouse lung

**Figure 1:**
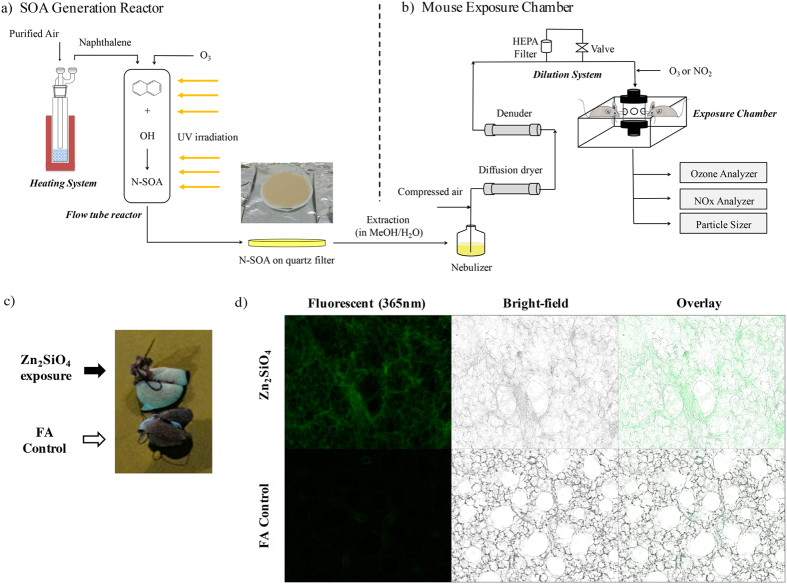
Atmospheric Simulation Reactor Exposure Platform (ASR) and exposure to Zn_2_SiO_4_ particles. The ASR is comprised of (**a**) a SOA generation reactor and (**b**) a mouse exposure chamber: (**a**) SOA is generated by photooxidation of organic vapor such as naphthalene in a quartz flow tube in the presence of ozone and UV, and collected onto quartz filters as shown. SOA is then nebulized, and passed through a diffusion dryer and a gas denuder to strip the aerosol of organic solvent (e.g. methanol) and water. (**b)** Delivery of SOA to the mouse exposure chamber is adjusted to the desired concentrations using a dilution system, and monitored in real-time using the scanning mobility particle sizer. O_3_ and/or NO_2_ are introduced downstream of the dilutor, and adjusted to desired concentrations. SOA with and without O_3_ and/or NO_2_ are delivered to the mice using the inExpose nose-only exposure chamber. (**c**) The lungs of mice exposed for 2 hr with test Zn_2_SiO_4_ particles (6 × 10^4^ particles/cm^3^; top image) or control filtered air (FA), explanted and immediately imaged under UV light (365 nm excitation wavelength), shows that the Zn_2_SiO_4_ particles (green), are diffusely distributed in all regions of the lung. Lungs from mice exposed to filtered air exhibit no green fluorescence (bottom image). (**d**) Lung slices of exposed mice were imaged using a fluorescence microscope, showing deposition of Zn_2_SiO_4_ particles (top panels) which fluorescence as green at excitation wavelength of 365 nm (far left panel) along with the walls of the alveoli (middle panel, bright-field image) in contrast to the control filtered air exposed lungs (bottom panels). Representative of 4 experiments.

The ASR developed in this study consists of two indispensable parts: an SOA generation reactor and a mouse exposure chamber, as shown in Fig. 1. The SOA generation reactor is coupled with ultraviolet (UV) light, and used to simulate atmospheric SOA formation from oxidation of various anthropogenic or biogenic precursors (Fig. 1a). SOA, collected on particle filters, was extracted into solution and nebulized for exposure. The second component is the nose-only inhalation exposure chamber (Fig. 1b), which allows for simultaneous exposure of 6–12 mice per experimental group to a uniformly distributed well-mixed stream of particle and gases. A dilution system allows direct control of concentrations (of SOA and gases) delivered for inhalation.

To characterize particle deposition efficiency in our ASR, we tested exposures of healthy mice using fluorescent indicator Zn_2_SiO_4_ particles. Zn_2_SiO_4_ aerosol was generated by a collision nebulizer with a concentration of 6 × 10^4^ particles/cm^3^ and number mode diameter of ~60 nm. After a 2 hr exposure, imaging of the whole lungs revealed uniform fluorescence (Fig. 1c), demonstrating effective and even deposition of the particles in the lung parenchyma. This was confirmed by fluorescence imaging of lungs from exposed mice with diffuse distribution of Zn_2_SiO_4_ beads (Fig. 1d, top panel) and control filtered air (FA)-exposed lungs which displayed no green fluorescence (Fig. 1d, bottom panel). Thus, we have shown our exposure apparatus to be a significant improvement over other exposure methods, such as intratracheal instillation which results in uneven particle exposure^16^,^19^.

### Well characterized SOA composition

**Figure 2:**
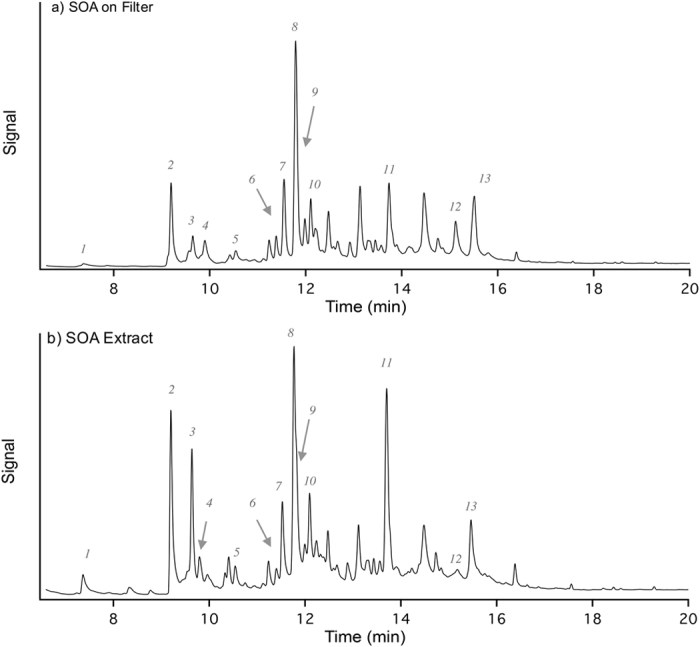
Total ion chromatogram of N-SOA analyzed using TD-GC/MS. (**a**) SOA on filter; (**b**) SOA extract with methanol/water solution. Comparisons of the chromatograms suggest that the extraction process does not significantly alter the structure/composition of SOA. The numbers shown on the chromatogram correspond to the components listed in Table 1.

N-SOA was generated in a quartz flow tube reactor through photooxidation of naphthalene, which simulates the major atmospheric fate for many hydrocarbons, particularly for PAHs^25^. The reactant flow rates were adjusted such that more than 90% of the initial naphthalene was consumed before SOA was collected for exposure. Chemical analysis using thermal desorption - gas chromatography mass spectrometry (TD-GC/MS) indicated that SOA constituents were consistent between on-filter SOA and extracted SOA (Fig. 2). The extracted N-SOA was used for the *in vivo* exposures.

A list of tentatively identified compounds is shown in Table 1. It is noted that peak #11 is tentatively identified as 2-formylcinnamaldehyde based on the calculated retention index and its fragmentation mass spectral pattern. Bunce *et al*.^25^ and Nishino *et al*.^26^, has previously shown that 2-formylcinnamaldehyde is a major product of naphthalene photooxidation in the gas phase. In this study, it is likely that 2-formylcinnamaldehyde partitions into the particle phase and observed on the SOA filters. Other major components observed include naphthoquinone, naphthalenol, naphthalenediol. These observations are consistent with previous chemical analysis using liquid chromatography-mass spectrometry^27^.

### Increasing methacholine responsiveness with increasing concentrations of N-SOA

**Figure 3:**
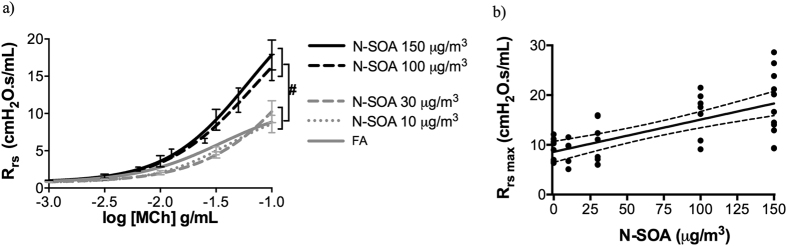
Healthy naïve BALB/c mice exhibit increased respiratory resistance to methacholine (MCh) following 3-day exposure to N-SOA. (**a**) The total lung resistance (R_rs_) to methacholine shows a dose-response relationship to increasing concentrations of N-SOA with the methacholine dose-responsive curves for N-SOA 100 μg/m^3^ and N-SOA 150 μg/m^3^ (^#^*p* < 0.05, n = 6–11/group). (**b**) The maximum resistance (R_rs_ max) showed significant dose-responsive increased to progressively higher concentrations of N-SOA (*p* < 0.05, r = 0.627, Spearman correlation, n = 6–11/group).

Daily exposure to N-SOA for three days significantly increased the responsiveness of the total respiratory system resistance (R_rs_) to methacholine (MCh) in an SOA dose-dependent manner when compared to filtered-air (FA) control. Differences in the MCh-dose responsive curves were significant between SOA concentrations at 100 and 150 μg/m^3^ and FA (Fig. 3a, #p < 0.05, n = 6–11/group). No significant differences were observed between the FA group and those exposed to SOA at 10 and 30 μg/m^3^. Differences between FA and the SOA at 100 or 150 μg/m^3^ were most obvious at the maximum R_rs_ with increasing concentration of N-SOA being significantly correlated to maximum R_rs_ (Fig. 3b, *p* < 0.05, r = 0.627, Spearman correlation).

### Additive effect of exposure to multi-pollutants

**Figure 4:**
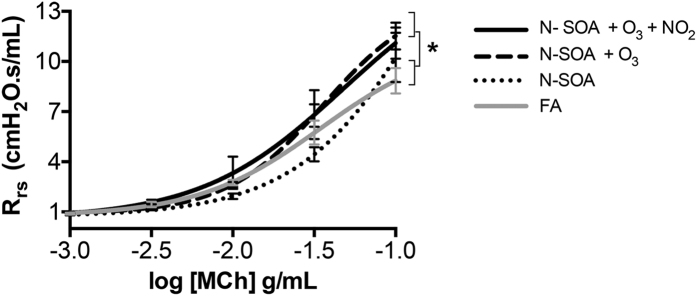
Co-exposure with O_3_ (65 ppb) ± NO_2_ (100 ppb) augments the respiratory response to N-SOA (30 μg/m^3^). The dose-response curves for R_rs_ to methacholine is significantly different for N-SOA + O_3_ and N-SOA + O_3_ + NO_2_ when compared with N-SOA alone or FA (*p < 0.05, n = 8–11/group).

To assess the potentiation of the airway responsiveness from exposure to multiple pollutants, we chose a concentration of N-SOA (30 μg/m^3^) that did not induce MCh-responsiveness in isolation, and conducted co-exposures with ambient concentrations of O_3_ (65 ppb), or O_3_ (65 ppb) + NO_2_ (100 ppb). Daily 1 hr exposure for 3 days to SOA + O_3_ or SOA + O_3_ + NO_2_ resulted in significant increases in the R_rs_ to MCh compared to FA controls. Differences were observed for the MCh dose-response curves (Fig. 4, *p < 0.05, dose-response curves n = 8–11/group) but not for the maximum R_rs_, suggesting that the effect of the co-exposures led to an increase in the sensitivity of the lung to MCh, rather than increasing the degree of airway contraction.

### No inflammatory cell recruitment after three days of exposure

**Figure 5:**
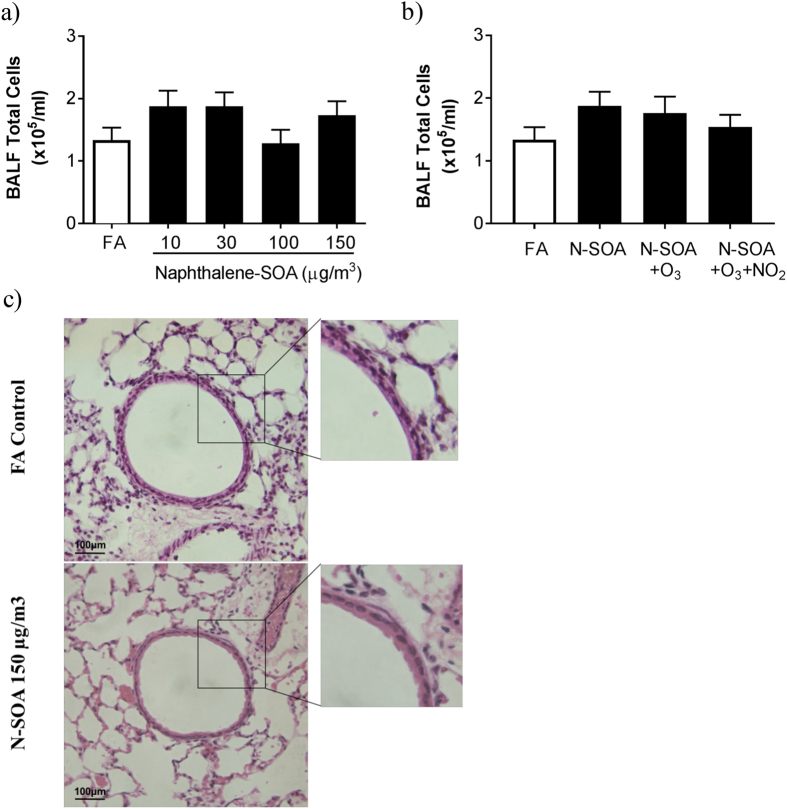
Exposure to N-SOA with/without O_3_ and/or NO_2_ did not result in inflammatory cell recruitment to the lungs. (**a**) Bronchoalveolar lavage fluid total leukocyte counts are similar in all exposure groups, regardless of concentrations of N-SOA or co-exposure with O_3_ and/or NO_2_ (*p* > 0.05, n = 5–11 mice/group). (**b**) Hematoxylin and eosin staining of lung sections from mice exposed to FA (top panel) and N-SOA (150 μg/m^3^, bottom panel) are similar and show no evidence of airway or lung inflammation.

Bronchoalveolar lavage fluid (BALF) total and differential cell counts were similar in all groups of mice including N-SOA exposures (Fig. 5a; *p* > 0.05, n = 5–11/group) and co-exposures with O_3_ and/or NO_2_ (Fig. 5b; *p* > 0.05, n = 5–11/group). Histological evaluation of the lungs from SOA-exposed were similar to the control FA-exposed, showing no inflammatory infiltrates or morphological changes (Fig. 5c). Together, these observations demonstrated that increased airway responsiveness to MCh was not associated with pulmonary inflammation, suggesting non-inflammatory pathways may be responsible for the responses observed.

## Discussion

In this study, we describe a novel atmospheric simulation reactor and exposure platform that allows repeated *in vivo* inhalation exposures of small animals to PM and gaseous pollutants at well-controlled concentrations. Coupled with the ability to conduct compositional analysis of the pollutants delivered for inhalation, this new tool permits mechanistic studies of *in vivo* models using experimental conditions that can be adjusted to mimic real-life chronic low-concentration multi-pollutant exposures. By introducing different PM and gaseous pollutants, and by altering the duration and intervals between exposures, our novel tool can be utilized to study different pollution exposure scenarios that best reflect human exposures in different times and at different geographic areas.

The main purpose of toxicological studies is to establish direct causal relationships between exposure to air pollutants and to elucidate cellular pathways activated by PM exposure that eventually lead to negative health outcomes. Toxicological studies provide the biological plausibility for epidemiological associations between PM and potential health effects^28^. Furthermore, they complement epidemiological studies by providing the understanding necessary to anticipate potential health implications prior to introduction of new air quality policies and/or emission reduction technologies^29^. The ASR developed in this work directly serves this purpose, as it is versatile and can be adapted to study effects of different pollutants. Besides N-SOA, other types of primary and secondary PM can also be generated in laboratory experiments to simulate exposure to PM from a variety of sources. In the midst of increasingly stringent emission standards and rapidly evolving mitigation technologies, the ASR can be easily adapted to characterize different emission sources and to rapidly address emerging health issues and develop risk mitigation strategies.

Here we have used the ASR to study the biological properties of SOA, which has been recognized over the past two decades to be an important component of ambient PM_2.5_^21^. While the chemical properties of SOA have received increased attention and advanced analytical techniques have been developed to investigate its complex composition^22^, the ASR adds a much-needed tool to characterize biological responses to *in vivo* chronic SOA exposures in animal models.

To date, the toxicity of SOA has been investigated mainly through *in vitro* studies^30^,^31^,^32^. Compared to the primary particles that are emitted directly from sources such as vehicular exhaust, SOA is more oxidized and is considered to have a higher oxidative potential^23^. It is postulated that pollutants with higher oxidative potentials *in vitro* generates greater oxidative stress *in vivo*, leading to adverse health outcomes^33^. A study from McDonald *et al*. suggests that *in vivo* oxidative stress may be related to SOA composition: A modest increase in expression of hemoxgenase (HO)-1 and matrix metalloproteinase-9 in the mouse aorta was observed following a 7-day exposure to α-pinene SOA in the presence of acidic aerosols^34^. However, exposure to toluene SOA or α-pinene SOA generated in the presence of neutral aerosols did not result in the same upregulation^34^,^35^. The composition of SOA is highly complicated and evolves dynamically upon atmospheric processing^36^. An added advantage of our experimental set-up is the capability to characterize SOA composition using analytical techniques such as GC/MS and LC/MS, which will allow us to link the aerosol composition to *in vivo* biological response.

Using the ASR, we are able to generate pollutant mixtures at defined concentrations and with reproducible composition, allowing us to conduct controlled studies and to build mechanistic insights into the PM-induced toxicological pathways. Here we observe that the degree of MCh-responsiveness following N-SOA exposure in healthy BALB/c mice is comparable to those observed in the chronic model of allergen–induced allergic airways inflammation, a model of human asthma^18^. However, in contrast to the allergic airways inflammation model, increases in the MCh-responsiveness in this experimental model were not accompanied by leukocyte recruitment to the airways or BAL fluid. Our observations are in agreement with previous mouse exposures studies from McDonald *et al*.^34^ using toluene SOA and α-pinene SOA generated in the presence of neutral aerosol. Farraj *et al*.^37^ also did not observe significant effects on inflammatory cell responses following exposures with diesel exhaust particles in allergic mice. *In vitro* studies, however, have suggested that SOA can induce inflammation^30–32^. Isoprene SOA increased inflammatory gene expression (interleukin-8 and cyclooxygenase-2) in human bronchial epithelial cells^30^, while α-pinene SOA decreased phagocytic activity in human macrophages, and trimethylbenzene SOA impaired epithelial wound repair^31^. The observed differences may reflect differences in the concentration and chemical composition of the specific SOA, as well as the experimental model (*in vitro* vs. *in vivo*)^31^,^34^, duration and route of delivery (inhalation vs. intranasal instillation)^16^,^19^, highlighting the need for experimental models that better reflect human exposures.

The major advantages of the ASR is the ability to deliver multiple pollutants at concentrations reflecting real-life ambient levels, as well as the ability to deliver these using a physiologically relevant route, i.e. inhalation, In other words, the ASR can recreate realistic exposure scenarios to support fundamental studies to assess causation and to elucidate the synergistic effects of multi-pollutant exposure. Ultimately, further development and deployment of this tool is an important step towards creating observations that are more readily translatable to human scenarios, and fully understanding the health impacts of air pollution.

## Methods

All animal protocols were approved by the University of Toronto Faculty Advisory Committee on Animal Services and conducted in accordance with the guidelines of the Canadian Council on Animal Care (CCAC).

### N-SOA generation and collection

Pure naphthalene solid (99%, Sigma-Aldrich, St. Louis, Missouri, USA) was heated to 55–65 °C in a glass tube, and the vapor was gently introduced into a quartz flow tube reactor in a 0.5 L/min flow of purified air. Ozone was produced by passing 1 L/min purified air through an ozone generator (No. 97006601, UVP, Cambridge, UK) and was photolyzed (λ = 180 nm) to produce singlet oxygen which further reacted with water vapor to generate hydroxyl radicals. Water vapor was produced by bubbling purified air through a custom-made humidifier. During naphthalene photooxidation, the inlet ozone concentration was maintained around 1 ppm. Reaction between hydroxyl radicals and naphthalene yielded N-SOA. The concentrations of naphthalene at the inlet and outlet of the flow tube were monitored with a gas chromatograph – flame ionization detector (GC/FID, Model 8610C, SRI Instruments Inc., Las Vegas, NV, USA) equipped with a Tenax® TA trap. Particle size distribution and mass concentration of N-SOA at the reactor outlet were continuously monitored using a scanning mobility particle sizer (SMPS) assembled in our lab, assuming a particle density of 1 g/cm^3^. The SMPS combines a long differential mobility analyzer column (Model 3081, TSI, Shoreview, MN, USA) with flow controls and a condensation particle counter (Model 3772, TSI, Shoreview, MN, USA). Data inversion to obtain size distribution was performed using custom code written in Igor Pro (Wavemetrics, Portland, OR, USA). In all experiments, reactor temperature (22–25 °C) and humidity (30–40% in flow tube) were monitored using an Omega HX94 C RH/T transmitter.

After the particle size distribution and volume concentration of N-SOA reached steady state, N-SOA was collected at the outlet of the flow tube onto 47 mm PTFE or prebaked quartz fiber filters (Pall, Ann Arbor, MI, USA) in a stainless steel filter holder. After collection, the filters were stored at −20 °C. Prior to animal exposure or chemical characterization, SOA was extracted using a 1:3 mix of HPLC grade methanol (99.9%, Sigma Aldrich, St. Louis, Missouri, USA) and Milli-Q water (18.2 MΩ · cm, Millipore Corporation, Billerica, MA, USA). Each quartz filter was sonicated at room temperature for at least 3 min in order to efficiently extract SOA into the solution.

### Chemical composition analysis of N-SOA

SOA composition was analyzed using a thermal desorption–gas chromatography mass spectrometry, or TD-GC/MS (TDS3, Gerstel, Mülheim an der Ruhr, Germany; Model 7890B and 5977A, Agilent, Santa Clara, CA, USA). Briefly, a small punch of SOA filter (containing about 13 μg SOA) was placed into a thermal desorption system and heated from 40 to 320 °C at 60 °C/min and then held at 320 °C for 5 min. Carried by helium gas, the desorbed organic compounds were trapped and re-concentrated in a cooled liner at 20 °C. The liner was then heated to 320 °C and kept at 320 °C for 10 min to transfer all the organic components to the GC/MS. An Rxi-5sil MS column with dimensions of 29 m × 250 μm × 0.25 μm (Restek Corporation, Bellefonte, PA, USA) was used during the measurements. GC oven temperature was ramped from 50 to 300 °C at 10 °C/min, and held at 300 °C for 5 min.

For the analysis of the SOA extract, the remaining filter was extracted in methanol/water (1:3) solution. After sonication and filtration (to remove of quartz debris), 10 μL of the extract (containing about 7 μg SOA) was added onto a punch of a blank prebaked quartz filter. The filter punch was then analyzed by the same method mentioned previously.

### Delivery of SOA and Zn_2_SiO_4_ particles to mouse lung

Naphthalene SOA or Zn_2_SiO_4_ (Sigma-Aldrich, St. Louis, Missouri, USA) particle solution was nebulized using a collision nebulizer (Model 3076, TSI, Shoreview, MN, USA). Water and methanol were removed from the particles by a custom-made diffusion dryer and a charcoal denuder (Aerosol Dynamics Inc., Berkeley, CA, USA), respectively (Fig. 1). A simple dilution system, with a filtered line (HEPA filter or charcoal filter and a metering valve) and a bypass line in parallel, was used to control the SOA concentration to within 50 μg/m^3^ of the desired concentration, which ranged between 10 and 150 μg/m^3^. The stream of particles was then mixed with ozone (produced by a UV ozone generator) and/or nitrogen dioxide, or directly entered the nose-only exposure chamber (Scireq Inc., Montreal, QC, Canada).

### Exposure protocol

Healthy 8–10 week old female BALB/c mice (Charles River Laboratories, Saint-Constant, PQ, USA) were used for all our studies.

To investigate the efficiency of particle delivery method, mice were exposed to either FA or Zn_2_SiO_4_ (6 × 10^4^ particles/cm^3^) for 2 hr. Lungs were immediately harvested, and imaged *en bloc* under UV light (365 nm excitation wavelength). In a subset of mice, the lung was infused with 1.2 mL of 5% low melt agarose-Hank’s balanced salt solution and cut into 150 μm thick slices using a tissue slicer (EMS-4000, Electron Microscope Sciences, Fort Washington, PA)^38^. Images were obtained using a ZEISS Fluorescence Microscope (Observer.Z1, Carl Zeiss Microscopy, Thornwood, NY, USA) with exposure times of 3000 ms and 80 ms for fluorescent (green, excitation wavelength of 365 nm) and bright-field image acquisition, respectively. An exposure duration of 1 hr yield similar observations and was thus used in the subsequent N-SOA exposures.

We chose N-SOA exposures of 1 hr/day to simulate the exposure duration to traffic related air pollutants of an average urban commuter. The exposures were conducted for 3 consecutive days. N-SOA (10, 30, 100, 150 μg/m^3^), ozone (65 ppb) and NO_2_ (100 ppb) were maintained at constant levels to mimic ambient levels. Particle size distribution, number and volume concentrations during exposures were monitored using SMPS. Ozone and NO_2_ concentrations were monitored using a UV photometric ozone analyzer (49i, ThermoScientific, Waltham, MA, USA) and NO-NO_2_-NO_x_ analyzer (42i, ThermoScientific, Waltham, MA, USA), respectively.

### Evaluation of lung function and methacholine responsiveness

Twenty-four hours after the last exposure, mice were anesthetized with ketamine (50 mg/kg i.p., Wyeth Animal Health, Guelph, ON, Canada) and xylazine (10 mg/kg i.p., Bayern Inc., Toronto, ON, Canada) to measure *in vivo* airway responsiveness to methacholine using the flexiVent^®^ system (Scireq Inc., Montreal, QC, Canada), as previously described^18^. Rocuronium (2 mg/kg i.p., Sandoz Canada Inc., Boucherville, QC, Canada) was administered during pulmonary function testing to prevent respiratory drive artifact. Baseline lung function was evaluated followed by assessment of methacholine responsiveness.

Immediately after assessment of lung function, mouse lungs were lavaged to assess the total BAL fluid leukocyte counts, as previously described^18^. In a subset of mice, lungs were harvested for histological analysis or stored at −80 °C.

### Histology of lung tissue sections

Lungs were extracted, inflated and embedded with 4% paraformaldehyde (Electron Microscopy Science, Hatfield, PA, USA) to a pressure of 25 cm H_2_O. Paraffin blocks were then prepared and sectioned (5 μm thickness) using Microm HM 325 (Microm GmbH., Walldorf, Germany). Slides were stained with Hematoxylin and Eosin (H&E) as previously described^18^, and imaged with inverted Leica DM IL microscope using 10x and 20x objectives and an Olympus DP71 camera.

### Statistical analysis

Data are expressed as the mean ± standard error of the mean (S.E.M.). Statistical analyses of pulmonary function data were carried out as described previously^18^. Methacholine dose-response curves of R_rs_ were compared using the F-test. 1-way ANOVA with post-hoc comparison with Dunn’s multiple comparison test were used for comparison between multiple groups. Differences were considered significant when *p* < 0.05. Statistical analyses were performed using Prism 6 (GraphPad Software Inc., La Jolla, CA, USA).

## Additional Information

**How to cite this article:** Ye, J. *et al*. Development of a Novel Simulation Reactor for Chronic Exposure to Atmospheric Particulate Matter. *Sci. Rep.* **7**, 42317; doi: 10.1038/srep42317 (2017).

**Publisher's note:** Springer Nature remains neutral with regard to jurisdictional claims in published maps and institutional affiliations.

## Supplementary Material

Supplementary Information

## Figures and Tables

**Table 1:** Summary of major N-SOA components measured by TD-GC/MS.

No.	Name	Structure	Molecular Weight (g/mol)	Retention Time (min)	Retention Index^a^ (measured)	Retention Index^b^ (literature)	MS Match^c^	MS Reverse Match^c^
1	Benzoic acid	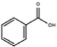	122.12	7.37	1167	1170	894	913
2	Phthalic acid	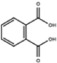	166.14	9.19	1299	n.a.^d^	977	976
3	1-Isobenzofuranone	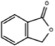	134.13	9.64	1333	n.a.	903	935
4	1,3-Indandione	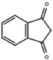	146.14	9.79	1344	1358	890	931
5	1,4-Naphthoquinone	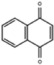	158.15	10.54	1402	1404	839	883
6	1,5-Naphthalenediol	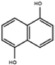	160.17	11.38	1469	n.a.	832	833
7	2-Acetylbenzoic acid	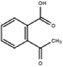	164.16	11.55	1483	n.a.	921	937
8	1-Naphthalenol	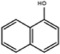	144.17	11.82	1505	1525	877	883
9	2-Naphthalenol	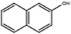	144.17	11.98	1518	1536	914	915
10	2-Hydroxy-1,4-naphthoquinone	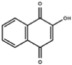	174.15	12.20	1537	n.a.	887	903
11	2-Formyl-cinnamaldehyde	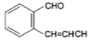	160.17	13.70	1666	n.a.	n.a	n.a
12	2,3-Naphthalenediol	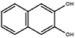	160.17	15.13	1799	n.a.	913	920
13	(3-Oxo-1,3-dihydro-2-benzofuran-1-yl)acetic acid	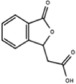	192.17	15.51	1836	n.a.	883	889

Compounds were identified by comparing measured retention indices to literature values, and/or by mass spectral matching. N-SOA composition revealed in this study is consistent with that measured previously^27^.

^a^Retention index was calibrated using *n*-alkane standard. Calculation of retention indices is illustrated in detail in Supporting Information.

^b^Reference literature for the retention indices of listed components can be found in Supporting Information.

^c^The fragmentation mass spectral patterns for SOA components were compared to the NIST EI-MS Library. Only peaks with Match and Reverse Match factors greater than 800 are listed.

^d^n.a. indicates that retention index is not available in the literature.
