# Supplementary material for: Development of a Novel Simulation Reactor for Chronic Exposure to Atmospheric Particulate Matter
Source: Sci Rep. 2017 Feb 7;7:42317. doi: 10.1038/srep42317 (PMC5294446; doi:10.1038/srep42317)
Supplement: Supplementary Information [file srep42317-s1.pdf]

**Supporting information:**

# Development of a Novel Simulation Reactor for Chronic Exposure to Atmospheric Particulate Matter

Jianhuai Ye[1], Sepehr Salehi[2], Michelle L. North[1], Anjelica M. Portelli[1], Chung-Wai Chow[1, 2, 3, *] and Arthur W. H. Chan[1, *]

[1] Department of Chemical Engineering & Applied Chemistry, University of Toronto
[2] Division of Respirology and Multi-Organ Transplantation Programme, University Health Network, University of Toronto
[3] Dalla Lana School of Public Health, University of Toronto

*Corresponding emails: arthurwh.chan@utoronto.ca or cw.chow@utoronto.ca

## Retention Indices of N-SOA Components

The retention index of a chemical compound is defined by normalizing its retention time to the retention times of co-eluting n-alkanes in the same gas chromatogram[1]. In temperature-programmed chromatography, the retention index of a certain compound $i$ can be calculated as:

$$I_i = \frac{t_i - t_n}{t_N - t_n} \times 100 + 100 \times n$$

where $I_i$ is the retention index of compound $i$; $t_i, t_n, t_N$ are the retention time of compound $i$, smaller n-alkane and larger n-alkane, respectively; $n$ is number of carbons in smaller n-alkane compound.

The references for the retention indices listed in Table 1 are shown as follows:

| Compounds | Reference |
|---|---|
| Benzoic acid | Ref. 2 |
| 1,3-Indandione | Ref. 3 |
| Naphthaquinone | Ref. 4 |
| 1-naphthalenol and 2-naphthalenol | Ref. 5 |

## References

1. Kováts, E. Gas-chromatographische Charakterisierung organischer Verbindungen. Teil 1: Retentionsindices aliphatischer Halogenide, Alkohole, Aldehyde und Ketone. Helv. Chim. Acta **41,** 1915–1932 (1958)
2. Alves, R.J.V., Pinto, A.C., da Costa, A.V.M., Rezende, C.M., Zizyphus mauritiana Lam. (Rhamnaceae) and the chemical composition of its floral fecal odor, *J. Braz. Chem. Soc.,* **16**, 654-656 (2005).
3. Rostad, C.E.; Pereira, W.E., Kovats and Lee retention indices determined by gas chromatography/mass spectrometry for organic compounds of environmental interest, *J. Hi. Res. Chromatogr. Chromatogr. Comm.,* **9**, 328-334 (1986).
4. Lopez-Avila, V.; Benedicto, J.; Baldin, E.; Beckert, W.F., Analysis of classes of compound of environmental concern: I. Nitroaromatic compounds, *J. Hi. Res. Chromatogr.,* **14**, 601-607 (1991).
5. Amorim, L.C.A.; Dimandja, J.-M.; Cardeal, Z. de.L., Analysis of hydroxylated polycyclic aromatoc hydrocarbons in urine using comprehensive two-dimensional gas chromatography with a flame ionization detector, *J. Chromatogr. A.,* **1216**, 2900-2904 (2009).
